# New insights into marine basin opening

**DOI:** 10.1093/nsr/nwz099

**Published:** 2019-08-05

**Authors:** Pinxian Wang

**Affiliations:** Professor, State Key Laboratory of Marine Geology, Tongji University, China Guest Editor of Special Topic

Break-up of continental lithosphere and opening of marine basins are one of the fundamental issues in geology, known as passive margin geodynamics. Its significance goes far beyond the academic interest as passive margin basins host nearly two-thirds of the discovered hydrocarbons worldwide. Our knowledge of the geodynamic evolution of passive margin originated largely from the northern Atlantic, resulting from multiple expeditions of international ocean drilling in the 1980s and 1990s. As a standard hypothesis of rifting and rupturing, the Atlantic model of passive margin has been applied widely to paleo- and modern basins from the Alps to Pacific; yet, its universal applicability needs to be tested by drilling in other basins, as recently done in the northern South China Sea (SCS).

From 2014 to 2018, three-and-a-half International Ocean Discovery Program expeditions (IODP 349, 367, 368 and 368X) took place in the SCS to explore its opening processes. With the motive of ‘testing hypotheses for lithosphere thinning during continental breakup’, 12 sites in total were drilled in the SCS deep basin with six sites recovering the igneous basement. The drilling results, however, negate the original predictions and challenge the prevailing wisdom.

Over the decades, the SCS has been considered as a nonvolcanic passive margin basin, similar to the Atlantic but with much smaller spatial and temporal scales. Using the Atlantic model as a template for interpretation of seismic data, a serpentinized mantle was proposed to exist at drilling sites along the continent-ocean transition zone in the northern SCS. Contrary to the expectations, however, the retrieval of mid-ocean ridge type basalt disproved the original assumption. The new drilling discoveries call for re-examination of the prevailing notions about the SCS opening, especially on its rifting and breakup mechanisms. Some of the most critical issues are being discussed in this special section.

The review paper by Wang *et al.* investigates the root of this misleading approach to the SCS geodynamics, and it emphasizes the distinction between two types of rifted basins: plate edge, such as the SCS, and intraplate, like the Atlantic. Separating these two types is necessary, because they appear to occur at different stages of the Wilson cycle with a contrasting style of lithospheric background.

Following the plate-edge rifting concept, Huang *et al.* introduce a new opening mechanism of the SCS, which underscores the role of the strike-slip faulting between the Eurasian plate and the relict Cretaceous Huatung plate east of the modern SCS. In this new model, the SCS developed first as a pull-apart basin by strike-slip motion before giving way to stepwise westward propagation of sea floor spreading.

The record of large scale volcanism and widespread magmatic underplating and diking in the SCS has led Sun *et al.* to suspect its compatibility with traditional model of magma-poor basins. Instead of taking on passive rifting like the Atlantic, these authors consider that the SCS breakup was strongly affected by plate subduction.

This idea further is supported by extensive postspreading volcanism revealed by ocean drilling. Lin *et al.* present new models of plate-induced mantle return flows, suggesting increased mantle upwelling beneath the SCS through interaction with surrounding subducting plates.

Jian *et al.* report the new discovery of deepsea Eocene sediments in the northern SCS, indicating deepwater conditions existing at the beginning of seafloor spreading, which was at least 5 Ma earlier than previously thought. This is again in sharp contrast to the Atlantic model, but it may feature the plate-edge rifting model.

Finally, the present-day extent of the SCS is merely one third of its area at the end of seafloor spreading. By reconstructing the entire SCS plate before eastward subduction, Zhao *et al.* investigate the interaction of the SCS with the Huatung Basin and Philippine sea plate.

Clearly, this special topic section is an attempt to challenge the universality of the prevailing concept of passive margin evolution in light of new findings from ocean drilling in the SCS. The proposed concept of plate-edge type is certainly far from mature, and the SCS geodynamics will remain a highly debated issue for years to come. Nevertheless, the ocean drilling discoveries presented here expose the contrasts between the Western Pacific and Atlantic, and they justify new visions and approaches toward revealing the geodynamic mysteries of the marginal basins.

